# The effect of anatomical variables and use of the Lifts system on hearing outcomes after implantation of an active transcutaneous bone conduction device in bilateral congenital conductive hearing loss

**DOI:** 10.1186/s40463-020-00452-3

**Published:** 2020-08-08

**Authors:** Jinsong Yang, Chunli Zhao, Yujie Liu, Mengdie Gao, Ran Ren, Danni Wang, Zhigang Huang, Shouqin Zhao

**Affiliations:** 1 Department of Otolaryngology and Head and Neck Surgery, Beijing Tong Ren Hospital, Capital Medical University, No 1, Dongjiaominxiang, Dongcheng District, Beijing, 100730 China; 2 Key laboratory of Otolaryngology and Head and Neck Surgery, Ministry of Education, 11th floor, no. 8, Chongwenmen Inner Street, Beijing, 100730 China

**Keywords:** 3D simulation, Transcutaneous bone conduction device, Hearing outcomes, Preoperative evaluation, Congenital microtia, Bonebridge

## Abstract

**Background:**

Malformations of the temporal bone present different challenges to the implantation of a transcutaneous active bone conduction device, such as Bonebridge (Med-el, Innsbruck, Austria). This study aims to describe the benefits of high-resolution computed tomography (HRCT) in preoperative assessment and to analyze whether characteristics of the mastoid process, intraoperative compression of the dura or sigmoid sinus, and the use of the Lifts system, lead to differences in audiological performance after implantation.

**Methods:**

We examined 110 cases of congenital microtia. The structure of the temporal bone was examined using HRCT and a 3D simulation software program. The mean anteroposterior mastoid bone thickness from the external auditory canal to the sigmoid sinus was measured (a measurement referred to as “AP”, hereafter). Sound field threshold (SFT), speech reception threshold (SRT) in noise, and word recognition score (WRS) in quiet, before and after implantation, were also measured. Independent variables were recorded in all patients: mastoid type (well pneumatized or poorly pneumatized), the presence of dural or sigmoid sinus compression, and the use of the Lifts system.

**Results:**

We found that the mean AP in the non-compression group was 16.2 ± 2.3 mm and in the compression group, 13.1 ± 2.9 mm (*p* < 0.001). We analyzed the hearing improvement of patients grouped by mastoid development, dural or sigmoid sinus compression, and use of the Lifts system, and found that these factors did not interact and that they had no influence on the hearing outcomes (*p* > 0.05).

**Conclusions:**

The AP dimension in the non-compression group was significantly larger than that in the compression group. This finding combined with the ROC curve analysis revealed the AP dimension was a high-accuracy predictor of potential surgical complications involving the dura and sigmoid sinus compression. Further analysis revealed that there was no interaction between the chosen variables: mastoid type, dural or sigmoid sinus compression, and the use of the Lifts system, and that all of these factors had no significant impact on hearing performance. Bonebridge was shown to produce effective and stable bone conduction and to improve patients’ hearing performance.

## Background

Malformations of the external ear (pinna or auricle and external auditory canal) are collectively termed microtia [1]. The reported prevalence varies among the different regions worldwide, from 0.83 to 17.4 per 10,000 births, and the prevalence is thought to be higher among Hispanics, Asians, Native Americans, and Andeans [2]. Congenital auricular atresia (CAA) is characterized by incomplete or failed development of middle ear structures and is often accompanied by microtia, atresia, and/or malformations of the auditory external canal [3]. In affected patients, a unilateral malformation is reported to be 3–5 times more common than bilateral, the latter seriously affecting the child’s speech and mental development [4, 5]. In the majority of cases, the inner ear structures are not affected [6]. Depending on severity, it causes a moderate to severe conductive hearing loss.

Hearing rehabilitation options for patients with CAA include canal tympanoplasty, a powerful air-conduction hearing aid, a bone-conduction device (BCD), or a middle ear implant (e.g. Vibrant Soundbridge, Med-el, Innsbruck, Austria) [7]. For patients with CAA who have a Jahrsdoerfer score ≥ 7, canal tympanoplasty used to be the treatment of choice [8, 9]. However, follow-up studies found that the postoperative sound field threshold (SFT) was in the range 25 to 35 dB HL, which was still equivalent to moderate conductive deafness. Approximately 30% of these patients needed conventional hearing aids to assist in hearing after surgery [10].

BCD can effectively improve hearing for patients with conductive or mixed hearing loss, transmitting vibrations directly to the cochlea via the bone of the skull. In transcutaneous active bone conduction devices, the transducer is implanted directly into the bone. The main advantage of BCD over conventional air conduction devices is that BCDs transmit vibrations through the skull directly to the cochleae, bypassing the external and middle ear, where the cause of the hearing loss lies [11].

There are different types of BCD. The earliest were active percutaneous devices such as BAHA Connect (Cochlear, Sydney, Australia) or Ponto (Oticon Medical, Vallauris, France). Though they produced good audiological outcomes, this kind of hearing aid came with significant drawbacks. Vibrations were transmitted via a screw rigidly anchored to the skull [12]. The screw had a diameter of 4.5 mm (narrow enough to be regarded as a single point of stimulation), and it passed directly from the bone, through the skin, creating a portal for infection. The implantation of the device was simple, but up to 37% of implanted children experienced at least one complication, mainly caused by the percutaneously implanted base [13]. Complications included recurrent soft tissue reactions and infections around the base (8 – 59%), implant loss (8.3%), and the need for additional surgery (5 – 42%) [14, 15].

Clearly a passive or active device which left the skin intact would have been preferable [16]. Passive devices, such as BAHA Attract (Cochlear, Sydney, Australia) and Sophono (Medtronic, Dublin, Ireland), transmit vibration through the skin and other tissues, tending to attenuate the energy of the vibrations. However, active devices such as Bonebridge (BB), generate vibration via a transducer which is attached directly to the skull, eliminating soft tissue attenuation issues.

The goal while placing the implant is to attach the transducer firmly to the skull in the best location, creating the best path for vibration, so that the level of stimulus reaching the cochleae is as high as possible. There are two main surgical approaches and techniques. The preferred approach is through the mastoid cavity region (under the middle cranial fossa, anterior to the sigmoid sinus, and posterior to the external auditory canal). The second is the retro-sigmoid approach. Most patients with CAA also suffer auricular, maxillofacial, and other abnormalities. These include temporal bone dysplasia or abnormal positions of the middle cranial fossa, the sigmoid sinus, or the external auditory canal. Further complicating treatment planning, the majority of patients are children whose skull cortex has not yet fully developed. Furthermore, BC-FMT is a relatively large transducer that is 8.7 mm in height, 15.8 mm in diameter and weighs 10 g. All these considerations need to be evaluated before BB implantation. Compared with the BAHA, BB implantation requires better skull development. Because of the relatively large size of the BB’s bone conduction floating mass transducer (BC-FMT), many studies have suggested using 3D simulation technology preoperatively to plan the optimal location for placing the implant [17]. For patients with a bone thickness of less than 8.7 mm or other abnormal temporal bone structure, the Lifts system can help avoid excessive pressure on the dura or sigmoid sinus. Dedicated BB Lifts have been developed by MED-EL and are available in 1, 2, 3, and 4-mm sizes [18]. The use of Lifts allows implantation in younger children, and facilitates implantation in difficult cases [19, 20]. Pre- and postoperative audiological tests are required to measure individual hearing improvement. Based on the surgical experience of the current authors, the key points to be considered for BB implantation are the degree of the patients’ malformation, the characteristics of the mastoid, the use of the Lifts system, and the presence of dural or sigmoid sinus compression.

Because of the wide variation in temporal malformations, there are many factors to consider in attaching and securing the transducer and its casing to the bone. Although BB implantation has already been demonstrated to be effective in practice, the influence of these different factors on audiological performance has not been investigated so far. This study aims to evaluate the audiological performance of BB in congenital microtia patients, in the presence of a number of different variables (the degree of mastoid pneumatization, intraoperative dural or sigmoid sinus compression, and the use of the Lifts system).

Specifically, we addressed the following questions:

1. Whether smaller antero-posterior (AP) dimension of the mastoid bone lead to intraoperative dural or sigmoid sinus compression.
2. Whether the characteristics of the mastoid to which the transducer is attached affect the patients’ postoperative audiological performance.
3. Whether intraoperative dural or sigmoid sinus compression affects the patients’ postoperative audiological performance.
4. Whether use of the Lifts system affects the patients’ postoperative audiological performance.
5. Whether the above factors interact with each other in the patients’ postoperative audiological performance.

## Methods

The study was approved by the Ethics Committee of the Institutional Ethics Committee of our hospital (No. Z171100001017079). We obtained informed consent from the patients included in this study.

### Subjects

This was an observational, single-center cohort study, including patients who received BB implantation for congenital microtia in the Otolaryngology Department of a tertiary-level referral hospital. Audiological evaluation results were obtained preoperatively, unaided, and compared to postoperative results with the BB activated. In China, parents often ignore hearing problems in children with unilateral congenital microtia, due to economic factors and the perceived poor aesthetics of hearing aids. All patients in this study had bilateral congenital microtia.

From April 20, 2016 to January 20, 2020, 110 patients implanted with BB (BCI 601, with the Amadé audio processor) were included in this study. The subjects had to meet the following inclusion criteria: 1) patients aged 5 years and above with congenital microtia; 2) patients with a skull thickness of ≥6 mm; 3) patients with a Jahrsdoerfer score ≤ 7, or with a Jahrsdoerfer score > 7, but not suitable for external auditory reconstruction or Vibrant Soundbridge implantation, due to stapes footplate fixation, tympanic cavity stenosis or oval window blockage by the facial nerve; 4) patients with bone conduction thresholds ≤45 dB HL, between 0.25 kHz and 4 kHz before implantation, with the difference between the two ears < 15 dB HL, to avoid eavesdropping; 5) at least 12 weeks of wearing the Amadé audio processor; 6) patients who understood Mandarin well, and were able to repeat words. Patients who did not meet the above criteria were excluded from the study. According to independent variables such as pneumatization of the mastoid, the presence of dural or sigmoid sinus compression, and the use of the Lifts system, patients were divided into several groups. All patients whose BC-FMT compressed 1 – 5 mm of the underlying soft tissues were classified as dural or sigmoid sinus compression.

### CT measurement

A large number of studies have shown that it is necessary to determine the implantation site of BC-FMT, by using imaging tools before surgery, as this can markedly reduce the surgery duration and risk [21–23]. “Fast View” software, developed by the Technical Research Center of the University of Navarra in Spain, was used in this study. Preoperative HRCT images were taken and the axial, coronal, sagittal and 3-dimensional simulation images, and BC-FMT images were analyzed. For successful BB implantation, it is recommended that compression of the dura mater, the sigmoid sinus, the temporomandibular joint and the external auditory canal be avoided as much as possible. To aid in deciding the best BC-FMT position, the AP was measured by two radiologists, independently. AP was measured on the axial CT slice in which the base of the cochlea first began to appear. A line was drawn from the base of the cochlea, perpendicular to the central axis. A second line was drawn, perpendicular to the first line, ending at the border of the sigmoid sinus. AP was defined as the length of this second line.

In 1940, Diamant was the first to report mastoid pneumatization in the literature. In all cases, mastoid lateral radiographs (Shuller position) were taken. According to the Diamant method, the geometric area of the mastoid aeration was first depicted on the X-ray film. This can be divided into two types: (1) well-pneumatized: the aeration area is larger than 6 cm^2^ and the air cell system is regular; (2) poorly-pneumatized: the aeration area is less than 6 cm^2^ and the air cell system is irregular. There is only partial aeration around the tympanic antrum or tympanic antrum entrance [24, 25].

### Surgical intervention and quality control

The BB implantation surgery was performed by, or supervised by, a senior doctor. The procedure is relatively simple and quick. There are three main approaches and techniques for placement. The most common method is via the mastoid; the second is via the retrosigmoid, and finally, the middle fossa [18, 26]. All operations were performed using the transmastoid approach in this study.

The transmastoid approach was performed as follows: access was gained via the retro-auricular sulcus; the BC-FMT bone bed was created, based on preoperative 3-dimensional simulation planning; after grinding BC-FMT bed, the bottom and sides of the bone bed were examined to determine whether there was an exposure of the dura or sigmoid sinus; fixation screw holes were drilled, and cortical screws inserted and tightened with a torque wrench (torque not exceeding 20 Nm). Depending on the intra-operative findings, the surgeon then decided whether the bone over the dura mater and/or sigmoid sinus had to be removed completely in order to adequately place the BC-FMT. In some cases, the temporal bone at the implantation site may be thin, and the use of the Lifts system is recommended [27, 28].

### Audiology evaluation methods

Pure tone audiometry (PTA), sound field thresholds (SFT), functional gain (FG), speech reception thresholds (SRT), and word recognition scores (WRS) were measured preoperatively, unaided, and the results were compared with postoperative, BB-aided results. The PTA was measured with a US GSI-61 audiometer (Grason-Stadler, Eden Prairie, MN, USA) to determine the air and bone conduction thresholds at 0.25, 0.5, 1, 2, and 4 kHz. For SFT measurement, the trill was presented from the front (S0) at 0.25, 0.5, 1, 2, and 4 kHz. SRT in noise (presented at 65 dB) was determined by an adaptive test method, with speech and noise coming from the front (S_0_N_0_). The result was expressed as signal-to-noise ratio (SNR) in dB, defined as the difference between speech presentation and noise level when the patient reached 50% speech recognition (SRT_50_). The WRS in quiet was measured by Mandarin Speech Test Materials (MSTM). Fifty monosyllabic words and fifty disyllabic words were presented at 65 dB SPL in quiet, and the percentage of correctly identified words was calculated .

### Statistical analysis method

All statistical analyses were performed with the SPSS 17.0 software. The Shapiro-Wilk test was used to test for normal distribution. Depending on the distribution, the paired t-test or Wilcoxon signed-rank test was used for comparison. To compare the efficacy of the device before and after implantation, the patients served as their own control. Multi-way ANOVA was performed to compare the postoperative benefits for each group. Furthermore, the predictor value of AP dimension for differentiating between dural or sigmoid sinus compression and non-compression was analyzed using receiver operating characteristic (ROC) curves. The ROC curve was plotted by calculating the sensitivity and specificity of the predictor to determine the best cut-off point. The optimal cut-off points were identified using the sensitivity, specificity, and ROC data. The diagnostic ability of each predictor was calculated based on the area under the curve (AUC), with an AUC value close to 1 indicating high predictability. An AUC value of > 0.9 was considered to represent high-accuracy, and AUC values of 0.7 – 0.9 and 0.5 – 0.7 represented moderate and low accuracy, respectively.

## Results

### Preoperative evaluation

The demographic information of the patients who underwent BB implantation is recorded. There were 110 patients with bilateral congenital microtia in the study (32 females and 78 males). They were 11.7 ± 5.2 years old (mean value [MV] ± standard deviation [SD]; range: 5.2 to 30.6 years). Fourteen patients had congenital aural stenosis and 96 patients, CAA. Five patients had a Marx grade I classification, and 105 patients had grade II or III [29]. The mean Jahrsdoerfer score for the 96 patients with CAA was 3.9 ± 2.1 (MV ± SD; range: 1.0 to 8.0 points). Sixty-three patients had a well-pneumatized mastoid and 47 patients were poorly-pneumatized. The BB was implanted in 68 patients without using the Lift system, in five with 1 mm Lift, 26 with 2 mm Lift, and 11 with 3 mm Lift. Sixty-seven patients had no dural or sigmoid sinus compression, and 43 patients had compression (dura: 18 patients, sinus: 14 patients, both: 11 patients). Lifts were used in 16 patients in the compression group. These Lifts could not prevent compression of the underlying soft tissues. The preoperative mean pure tone bone conduction threshold (PTA_5_: 0.25, 0.5, 1, 2, and 4 kHz) at the implanted side was 8.4 ± 6.2 dB HL (range: − 5.0 to 32.0 dB HL). The preoperative mean air conduction threshold at the implanted side was 66.9 ± 7.8 dB HL. The average usage time of the Amadé audio processor, from activation to postoperative testing, was 25.6 ± 6.3 weeks (range: 12 to 38 weeks).

### Measurement of the mastoid

**Fig. 1:**
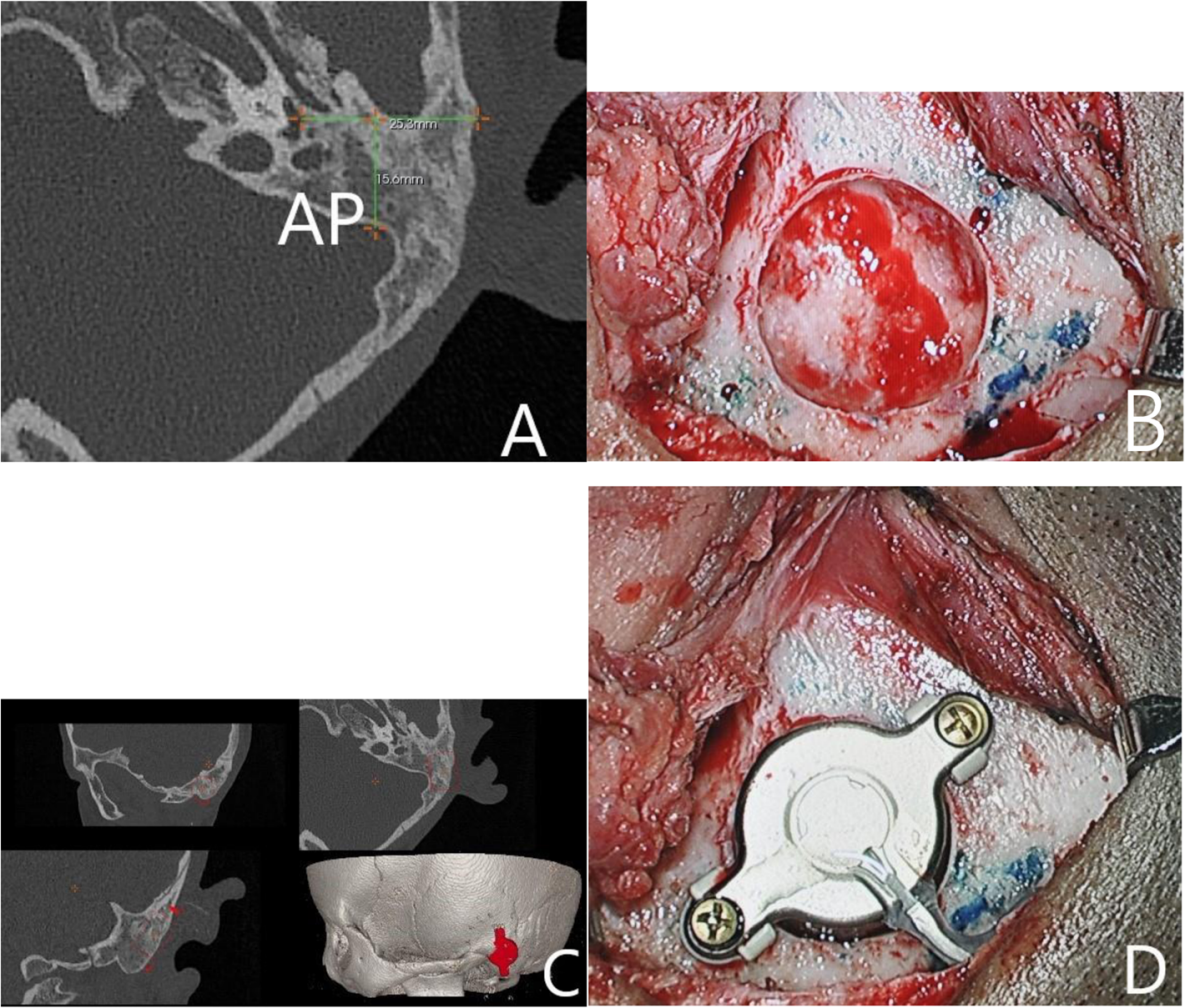
A patient belonging to Group-nocom. **a** The length of AP. **b** Preparation of BC-FMT bone bed based on preoperative 3D simulation positioning. **c** Preoperative HRCT three-dimensional simulation images. **d** The implanted BC-FMT with Lifts. BC-FMT, bone conduction floating mass transducer; HRCT, high-resolution computed tomography

**Fig. 2:**
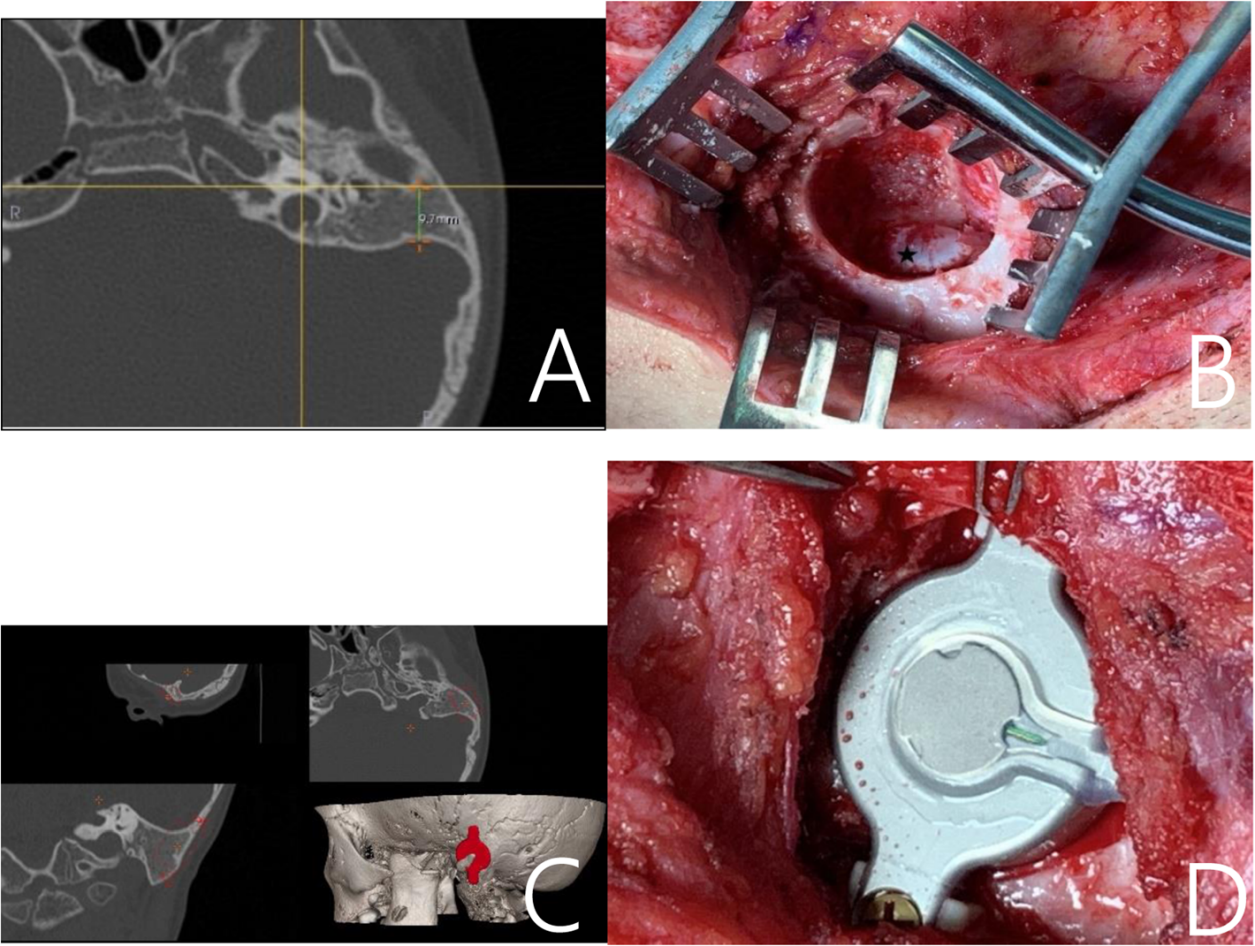
A patient of Group-com. **a** The length of AP. **b** Preparation of BC-FMT bone bed based on preoperative 3D simulation positioning. **c** Preoperative HRCT three-dimensional simulation images. **d** The implanted BC-FMT with Lifts. * denotes site of sigmoid sinus exposure. BC-FMT = bone conduction floating mass transducer; HRCT = high-resolution computed tomography

**Fig. 3:**
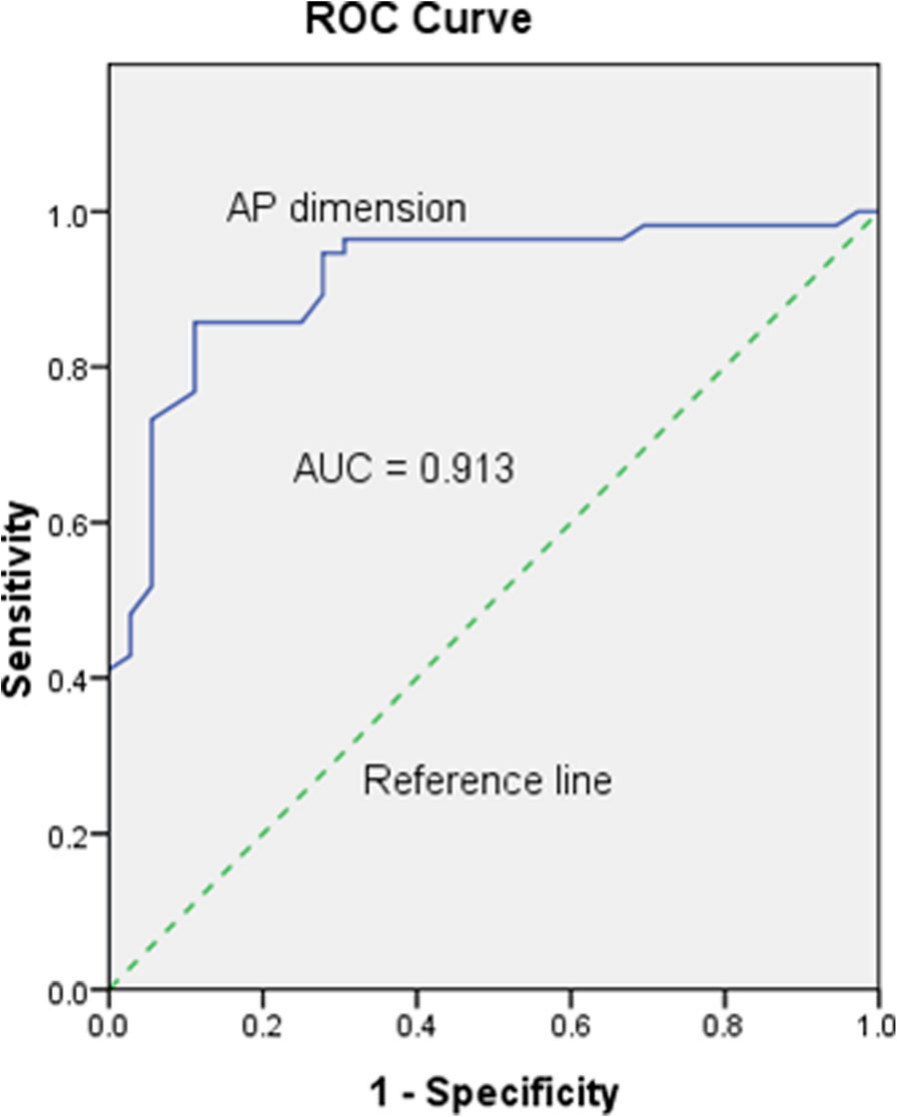
ROC curves of AP dimension. The AP had high accuracy for (AUC = 0.947) dural or sigmoid sinus compression (AUC = 0.913). AUC = area under the receiver operating characteristic curve; ROC = receiver operating characteristic; AP = the mean antero-posterior mastoid bone thickness from the external auditory canal

During surgery, 43 patients had compression of the dura or sigmoid sinus (Group-com), and 67 patients did not (Group-nocom). We measured the length of AP in preoperative HRCT with 3D simulation software, retrospectively. The mean AP of Group-nocom was 16.2 ± 2.3 mm (Fig. 1) and of Group-com, 12.9 ± 2.8 mm (Fig. 2). There was a significant difference between the two groups (*p* < 0.001). ROC curve analysis of the AP dimension associated with compression is presented in Fig. 3. The best cut-off point for AP dimension was determined using the Youden index to differentiate dural or sigmoid sinus compression. We found that a value of 14.35 mm for E/M ratio resulted in a Youden index of 74.6%, with a sensitivity of 85.7% and a specificity of 88.9%. The AP dimension showed high accuracy as a predictor for dural or sigmoid sinus compression (AUC = 0.913).

### Pure tone audiometry

In our previous study, BB implantation was shown to be a safe and effective method to significantly improve audiological and subjective benefits of patients [30–32]. In this study, the mean, preoperative, pure tone, bone-conduction threshold (PTA_5_) was 8.4 ± 6.2 dB HL (range: − 5.0 to 32.0 dB HL). This did not change significantly at first activation after surgery, with a mean of 8.5 ± 5.2 dB HL (range: − 2.0 to 28.0 dB HL; *p* > 0.05, paired t-test). Furthermore, after surgery, bone conduction did not significantly change at any frequency (*p* > 0.05, t-test).

**Fig. 4:**
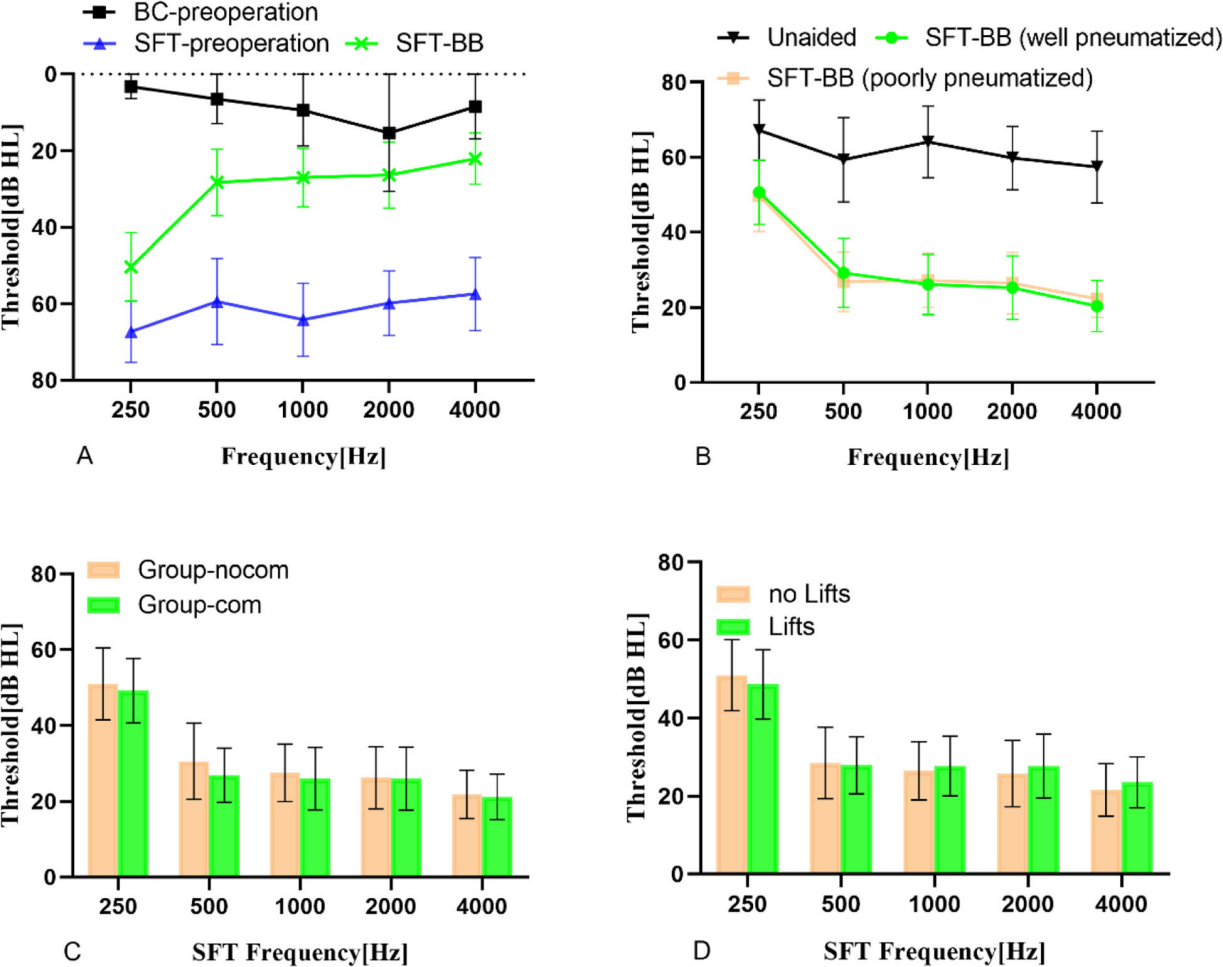
**a** Preoperative bone conduction and SFT performance before and after BB implantation. **b** SFT performance unaided, and with BB, in different mastoid types. **c** SFT performance with BB, with and without dural or sigmoid sinus compression. **d** SFT performance with BB, with and without Lifts system use. Error bars depict standard deviation

We analyzed the hearing improvements of patients, grouped by mastoid type, dural or sigmoid sinus compression, and use of the Lifts system, with multi-way ANOVA, and found that these variables had no influence on SFT improvement. We found a significance between the mean SFT results (SFT_5_) under the unaided and BB aided conditions (Fig. 4a; paired t-test, *p* < 0.001). However, mastoid type, presence of dural or sigmoid sinus compression, and use of the Lifts system did not affect SFT results (Fig. 4; *p* > 0.05). The FG result in SFT_5_ for the well-pneumatized mastoid group was 32.2 ± 9.6 dB HL and for the poorly-pneumatized group was 32.3 ± 8.7 dB HL (Fig. 4b; *p* > 0.05). The FG result in SFT_5_ for Group-nocom was 31.9 ± 10.2 dB HL, whereas that for the Group-com was 32.7 ± 7.3 dB HL (Fig. 4c; *p* > 0.05). The FG result in SFT_5_ for the group using Lifts was 32.4 ± 8.0 dB HL and for the group without Lifts was 32.1 ± 9.9 dB HL (Fig. 4d; *p* > 0.05).

### Speech audiometry

**Fig. 5:**
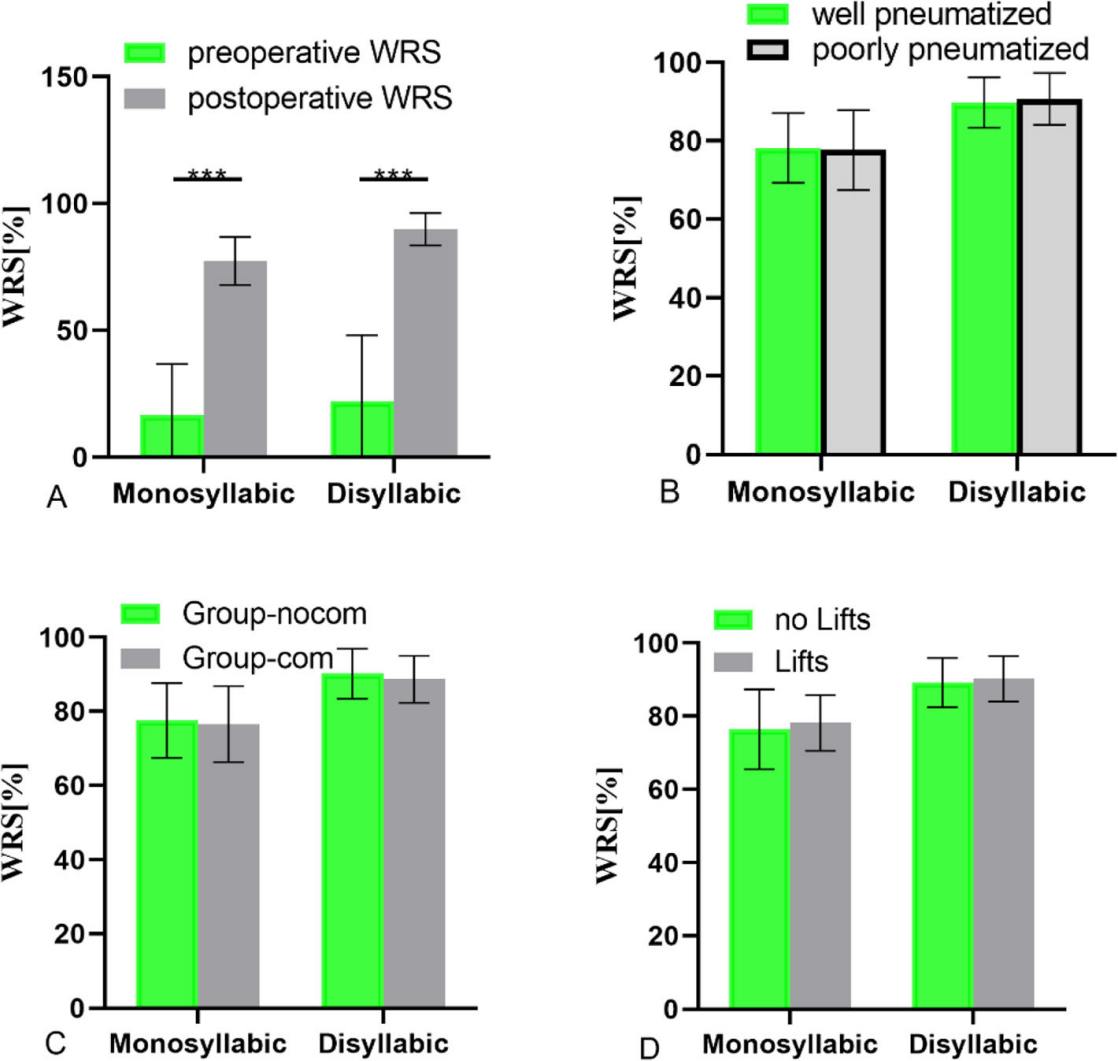
**a** The comparison of WRS results (monosyllabic and disyllabic words), before and after BB implantation. **b** The WRS results of different mastoid types. **c** The comparison of WRS results, with and without dural or sigmoid sinus compression. **d** The comparison of WRS results, with and without Lifts system use. Error bars depict standard deviation

A comparison of results under the unaided and BB-aided conditions found that the BB-aided performance was significantly better (Fig. 5a; *p* < 0.05, paired t-test), and monosyllabic words in WRS improved more than disyllabic (*p* < 0.05). However, mastoid type, presence of dural or sigmoid sinus compression, and use of the Lifts system did not affect the WRS results (Fig. 5; *p* > 0.05). The monosyllabic WRS score of the well-pneumatized mastoid group was 78.1 ± 9.4 and of the poorly-pneumatized group, 78.8 ± 9.5 dB HL. The disyllabic WRS score of the well-pneumatized mastoid group was 90.8 ± 6.4 and of the poorly-pneumatized group was 89.6 ± 6.1 dB HL (Fig. 5b; *p* > 0.05). The monosyllabic WRS score of Group-nocom was 78.0 ± 9.6 dB HL and of Group-com was 79.0 ± 9.3 dB HL. The disyllabic WRS score of Group-nocom was 90.8 ± 6.5 dB HL and of Group-com was 89.6 ± 5.9 dB HL (Fig. 5c; *p* > 0.05). The monosyllabic WRS score of the group without Lifts was 78.2 ± 10.5 dB HL and of the group using Lifts was 78.7 ± 7.7 dB HL. The disyllabic WRS score of the group without Lifts was 90.1 ± 6.3 dB HL and of the group using Lifts was 90.6 ± 6.3 dB HL. There was no significance difference in WRS with or without the Lifts system, in monosyllabic or disyllabic words. (Fig. 5d; *p* > 0.05).

**Fig. 6:**
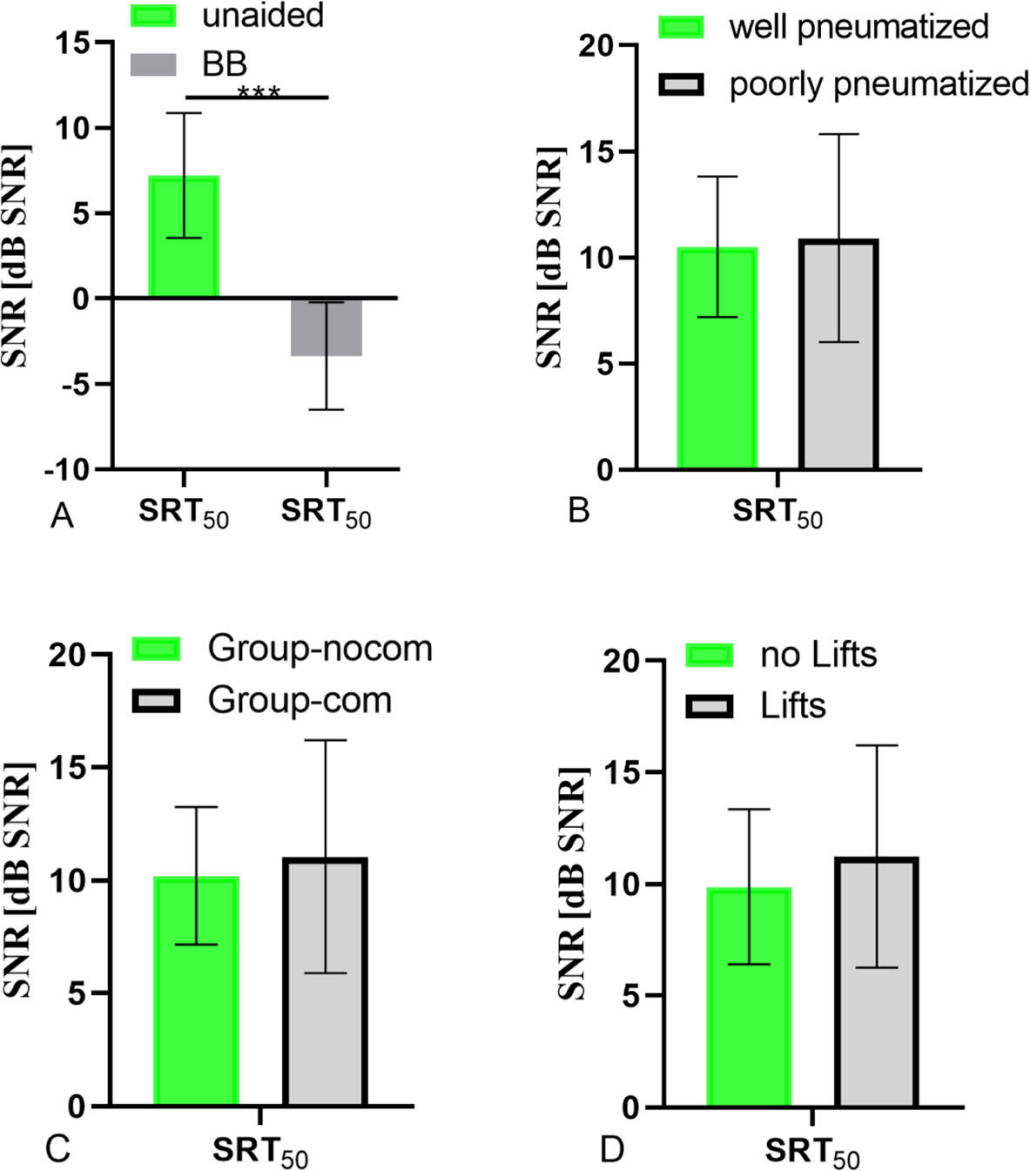
**a** The comparison of SRT results, before and after BB implantation. **b** The SRT results of different mastoid types. **c** The comparison of SRT results, with and without dural or sigmoid sinus compression. **d** The comparison of SRT results, with and without Lifts system use. Error bars depict standard deviation

After the analysis of the SRT results, the SNR values clearly showed a significant improvement following BB implantation (Fig. 6a; paired t-test, *p* < 0.05). The mean FG result (in SNR) in the well-pneumatized mastoid group was 10.1 ± 3.2 dB and in the poorly-pneumatized group was 10.9 ± 5.0 dB (Fig. 6b; *p* > 0.05). The mean FG result (in SNR) in Group-nocom was 10.5 ± 4.7 dB, and in Group-com, 10.3 ± 2.8 dB (Fig. 6c; *p* > 0.05). The mean SNR improvement of the group without Lifts was 10.1 ± 4.7 dB, and of the group using Lifts, 10.9 ± 2.7 dB (Fig. 6d; *p* > 0.05).

### Complications

At present, no adverse events were found during the follow-up of this study, and none of the patients experienced flap necrosis, infection around the operative area, hearing deterioration, or implant rejection. There was no increase in postoperative headache due to direct stimulation of the soft-tissue structures.

## Discussion

Unilateral and bilateral permanent hearing loss have long been known to put children at risk of learning difficulties. Most patients with congenital microtia have some sort of temporal bone dysplasia, which presents challenges for BC-FMT implantation. Surgery may be complicated by a variety of anatomical anomalies, such as malformation of the mastoid, the position of the lower sigmoid sinus, or an abnormal external auditory canal. For this reason, planning is key. A large number of studies have shown that it is helpful to determine the implantation site for BC-FMT, in advance, by using imaging tools. To reduce surgery duration and risk in our study, the BB Fast View program was used to simulate the implantation of BC-FMT, and evaluate the possibility of sigmoid sinus compression, the mastoid type, and the potential need for the Lifts system. To our current knowledge, this is the first study to systematically evaluate the hearing outcomes of patients with BB, in relation to the aforementioned three common surgical issues, and to determine whether their interaction influences the outcomes.

As the mastoid volume increases with age, the probability of the BC-FMT fitting a child, increases. This is helped, in particular, by the increasing thickness of the mastoid in the sinus-dura angle, the qualitative change in the bone, and the improvement in the mastoid shape between the ages of 3 and 6 years, as indicated by accelerated growth of the mastoid tip. However, children with congenital microtia may have a smaller mastoid [19]. We retrospectively measured AP, using preoperative HRCT and 3D simulation software. Patients with intraoperative dural or sigmoid sinus compression were found to have had a smaller mastoid volume (a shorter AP). Our findings reveal that it is uncommon for BB candidates who have CAA, to have a sufficiently capacious mastoid bone to accommodate the BC-FMT entirely, because of mastoid hypoplasia. Due to the large size of BC-FMT, and the shorter length of AP in these patients, intraoperative compression is more likely. Thus, AP may be used as a parameter for preoperative evaluation, to remind the surgeon of the possibility of dural or sigmoid sinus exposure during preparation of the bone bed for BC-FMT. Eric K, et al. found the use of Lifts, or sigmoid sinus or dural depression, necessary to accommodate the BC-FMT in the majority of cases, including most mastoid placements [33].

Our study showed that all patients with BB obtained obvious audiological benefits. You Chang, et al. found that the vibration response at the cochlear promontory was similar, at frequencies below 0.5 kHz, with all BCDs. At higher frequencies, above 4 kHz, direct-drive BCDs show the greatest cochlear promontory vibration response [11]. Our study also showed that SFT outcomes are better at higher frequencies. To address safety concerns, the BB manufacturer introduced an accessory called the Lifts system, enabling the surgeon to reduce the insertion depth. Originally, we thought that there might be an interaction between the three chosen factors (mastoid pneumatization, dural or sigmoid sinus compression and the use of the Lifts system) in influencing postoperative hearing outcomes. However, our results show no such interaction. Furthermore, the presence or absence of these factors did not affect audiological outcomes in SFT, WRS or SRT. Patients who received additional, non-osseous stimulation via the dura or the sigmoid sinus, showed no statistically significant differences in SFT, WRS and SRT values, compared to the group with only bone conduction stimulation. Previous studies also found that direct stimulation of the soft tissue structures within the skull, by a transcutaneous bone conduction implant, provides satisfactory hearing outcomes [34]. Our further analysis found that different degrees of mastoid pneumatization did not interfere with hearing sensation. We further studied the effect of the Lifts system on audiological outcomes, and found that the use of Lifts, even if the BC-FMT lift was moderately high, did not affect hearing sensation.

## Conclusions

Considering the complexity of temporal bone malformation, careful CT evaluation using 3D software for precise device simulation plays an important role in reminding surgeons of the dangers of encountering intraoperative dural or sigmoid sinus exposure and evaluating whether Lifts should be used. The AP dimension in the non-compression group was significantly larger than that in the compression group. This finding combined with the ROC curve analysis revealed the AP dimension is a high-accuracy predictor of potential surgical complications involving the dura and sigmoid sinus compression. Further analysis revealed that there was no interaction between the chosen variables: mastoid type, dural or sigmoid sinus compression, and the use of the Lifts system, and that all of these factors had no significant impact on hearing performance. BB was shown to produce effective and stable bone conduction and to improve hearing performance patients with microtia.

## Data Availability

Not applicable.

## Abbreviations

HRCT: High-resolution computed tomography
SFT: Sound field threshold
SRT: Speech reception threshold
WRS: Word recognition score
AP: The mean anteroposterior mastoid bone thickness from the external auditory canal to the sigmoid sinus
CAA: Congenital auricular atresia
BCD: Bone-conduction device
BB: Bonebridge
BC-FMT: Bone conduction floating mass transducer
PTA: Pure tone audiometry
FG: Functional gain
SNR: Signal-to-noise ratio
ROC: Receiver operating characteristic
AUC: Area under the curve
